# Supplementary material for: Drosulfakinin signaling in fruitless circuitry antagonizes P1 neurons to regulate sexual arousal in Drosophila
Source: Nat Commun. 2019 Oct 18;10:4770. doi: 10.1038/s41467-019-12758-6 (PMC6800437; doi:10.1038/s41467-019-12758-6)
Supplement: Supplementary file 1 — Supplementary Information [file 41467_2019_12758_MOESM1_ESM.pdf]

Drosulfakinin signaling in *fruitless* circuitry antagonizes P1 neurons to regulate sexual arousal in *Drosophila*

Wu et al.

Supplementary Information

Supplementary Figures and Legends

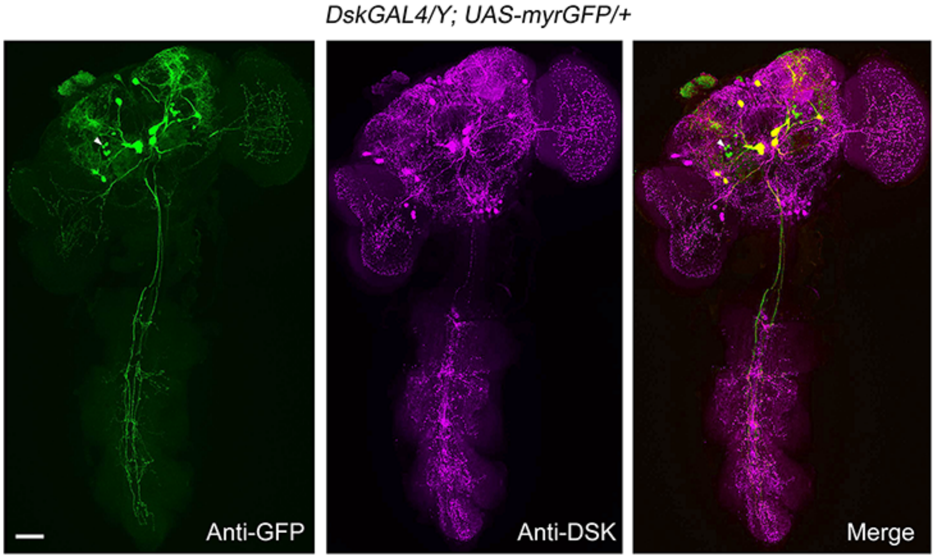

Supplementary Fig. 1 Expression pattern of the *DskGAL4* (X chromosome) in the central nervous system. This GAL4 line was only used during behavioral screen (Fig. 1b). Arrowhead indicates non-*Dsk* neurons labeled by this *DskGAL4*. Scale bars, 50 µm.

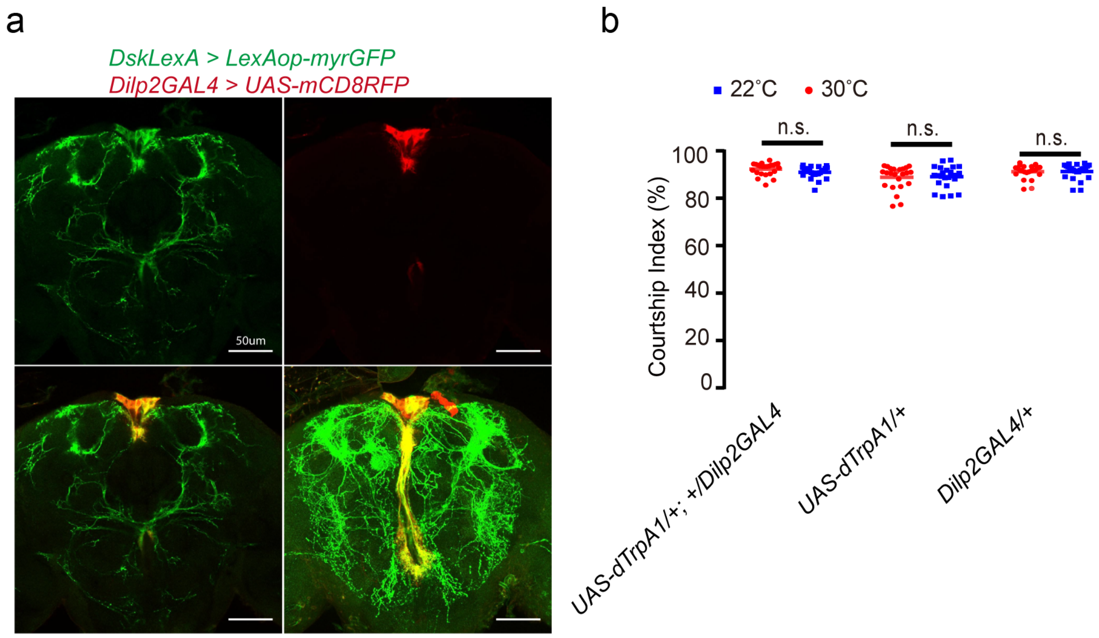

Supplementary Fig. 2 *Dsk*-positive insulin-producing cells are not responsible for courtship inhibition. **a** Co-labeling of insulin-producing cells in the Pars

Intercerebralis (PI) region by *DskLexA* (green) and *Dilp2GAL4* (red). Representative of 5 male brains. Scale bars, 50 µm. The bottom right panel indicates Z-projection of the full stack. **b** Activating *Dsk*-positive insulin-producing cells alone labeled by *dilp2GAL4* does not affect male courtship. $n = 24$ for each. $p < 0.001$, Kruskal-Wallis test. $p = 0.6481$, $p > 0.9999$, $p > 0.9999$ respectively, post hoc Dunn's multiple comparisons test. n.s., not significant. Error bars indicate SEM. Source data are provided as a Source Data file.

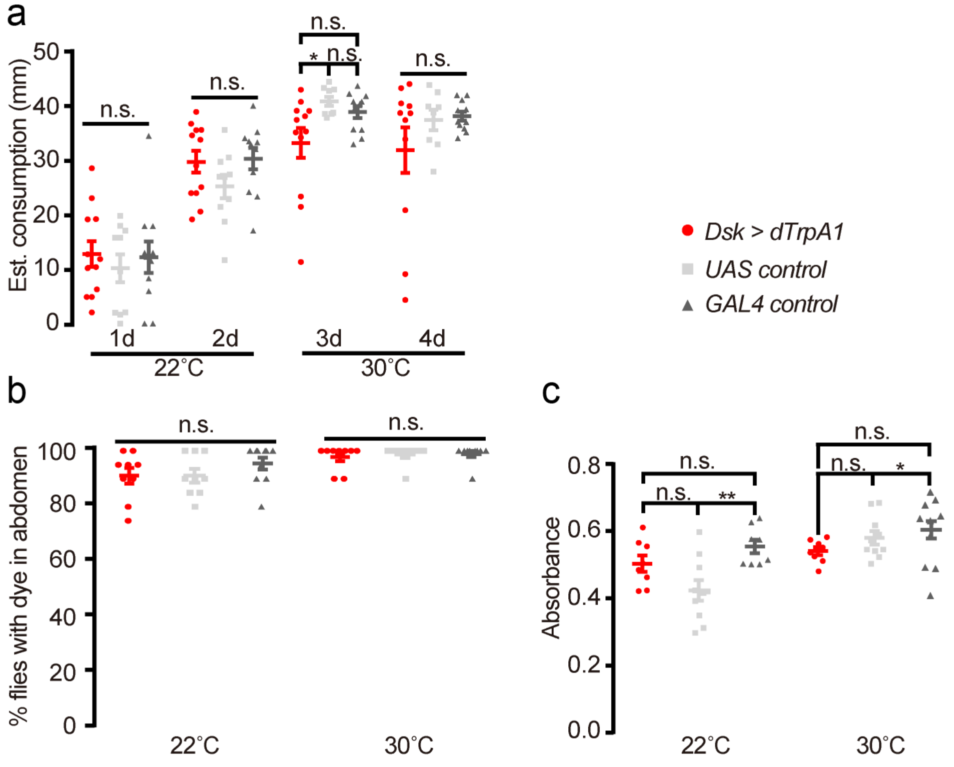

Supplementary Fig. 3 Activating *DskGAL4* neurons does not affect feeding behavior. **a** Activation of *Dsk* neurons in males has no effect on food intake as measured by CAFE assay for 4 consecutive days. Day 1: n =12, 10 and 11, $p = 0.7673$, one-way ANOVA. Day 2: n =12, 10 and 11, $p = 0.1839$, one-way ANOVA. Day 3: n =12, 9 and 11, $p = 0.0639$, one-way ANOVA. Day 4: n =11, 8 and 11, $p = 0.2382$, one-way ANOVA. **b** Ingested food with blue dye was visually counted in the abdomen of

starved male flies after 15-min feeding. 22˚C: n = 9, 9 and 10, p = 0.2880, Kruskal-Wallis test. 30˚C: n = 9, 8 and 9, p = 0.7861, Kruskal-Wallis test. Each sample contains 20 males. **c** The absorbance of ingested colored food was measured in starved male flies after 15-min feeding. 22˚C: n = 8, 10 and 8, p = 0.0054, one-way ANOVA, **p = 0.0045, p = 0.0955 and 0.4008 (n.s.) respectively, post hoc Tukey's multiple comparisons test. 30˚C: n = 8, 10 and 10, p = 0.0346, one-way ANOVA, *p = 0.0266, p = 0.4051 and 0.4007 (n.s.) respectively, post hoc Tukey's multiple comparisons test. Each sample contains 20 males. n.s., not significant. Error bars indicate SEM. Source data are provided as a Source Data file.

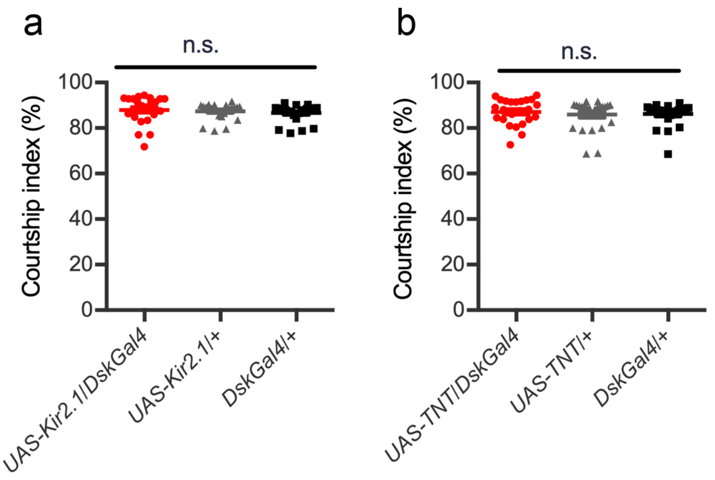

Supplementary Fig. 4 Inhibiting *DskGAL4* neurons does not affect male courtship. **a**, **b** Blocking *DskGAL4* neuronal transmission using Kir2.1 (**a**) or tetanus toxin (TNT) (**b**) does not affect male courtship to wild-type virgin females. n = 24 for each. For Kir2.1: p = 0.1650, Kruskal-Wallis test. For TNT: p = 0.3673, Kruskal-Wallis test. n.s., not significant. Error bars indicate SEM. Source data are provided as a Source Data file.

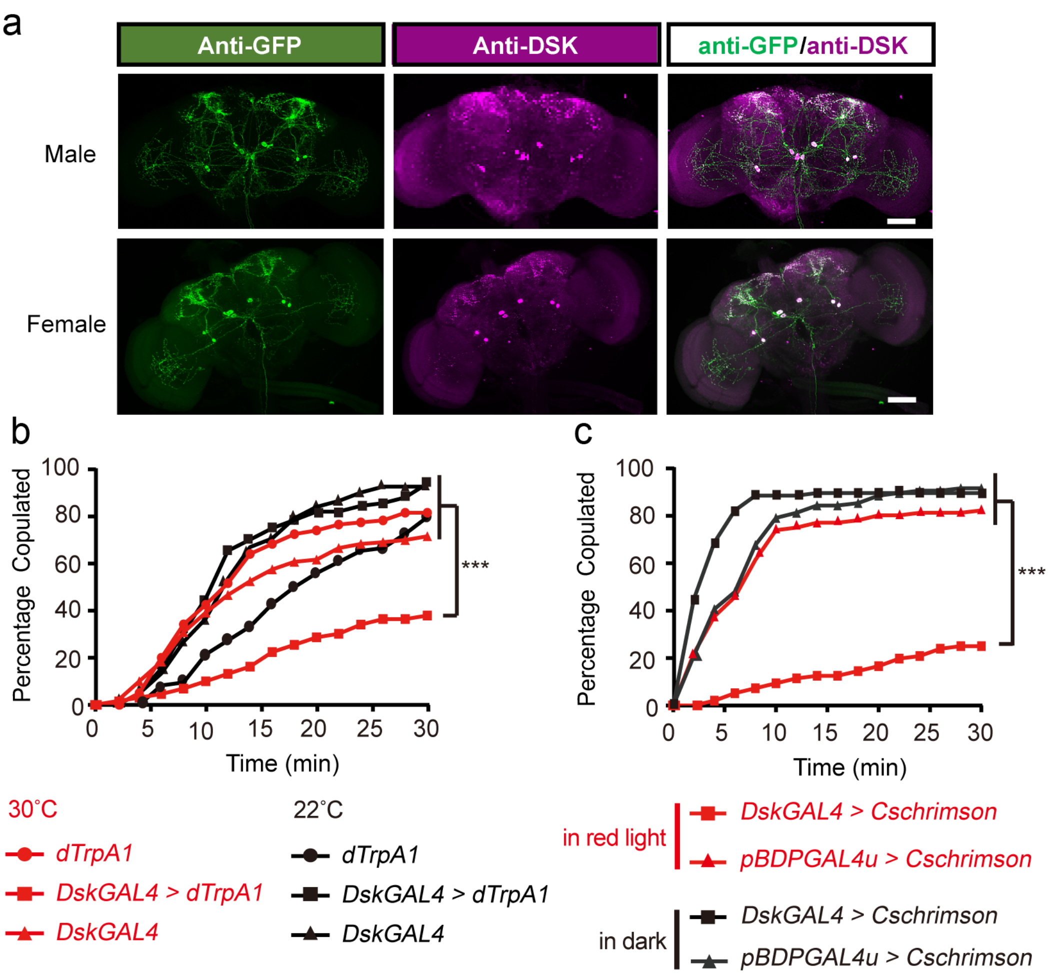

Supplementary Fig. 5 *Dsk* neurons are sexually monomorphic and inhibit male and female sexual behaviors. **a** There is no obvious *Dsk* expression difference between male and female. Representative of 8 males and 5 females. Two pairs of MP1 and two pairs of MP3 neurons are labeled in all samples. Scale bars, 50 μm. **b** Thermogenic activation of *DskGAL4* neurons reduces female receptivity. n = 82~120 for each. ***p < 0.001, chi-square test. **c** Optogenetic activation of *DskGAL4* neurons impairs female receptivity. n = 60~84 for each. ***p < 0.001, chi-square test. Source data are provided as a Source Data file.

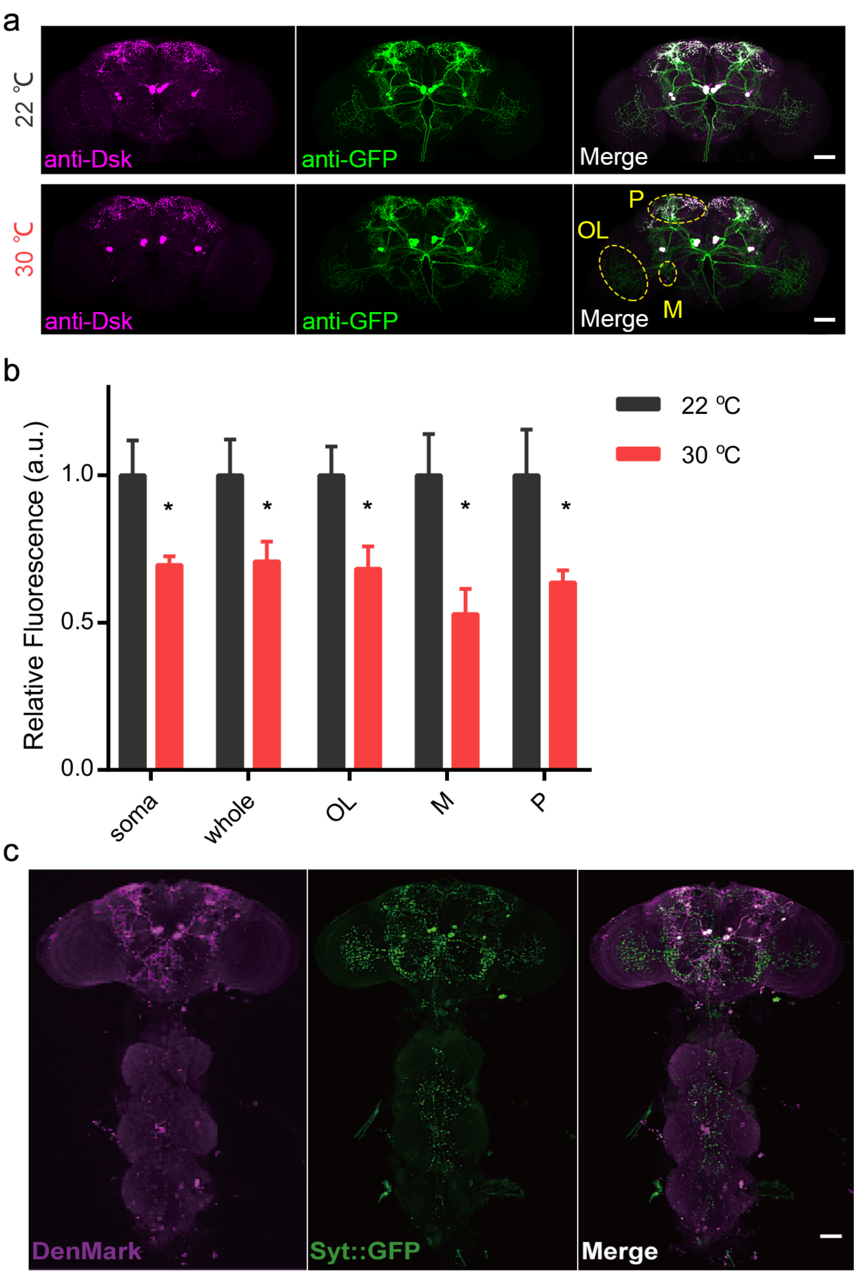

Supplementary Fig. 6 Activating *Dsk* neurons induces secretion of DSK peptides. **a** Examples of co-staining of DSK and GFP in *Dsk* neurons with or without *dTrpA1* activation (30 min heat treatment at 30˚C). **b** Activating *Dsk* neurons for 30 min reduces DSK retention in soma, optic lobes (OL, mainly presynaptic), median (M) and protocerebrum (P), possibly by secreting DSK. n = 7 and 10 respectively for 22˚C and 30˚C. *p <0.05, two-tailed unpaired t test. **c** Pre-synaptic and post-synaptic labeling of *Dsk* neurons by syt::GFP and Denmark, respectively. Representative of 4

male flies. Scale bars, 50 μm. Source data are provided as a Source Data file.

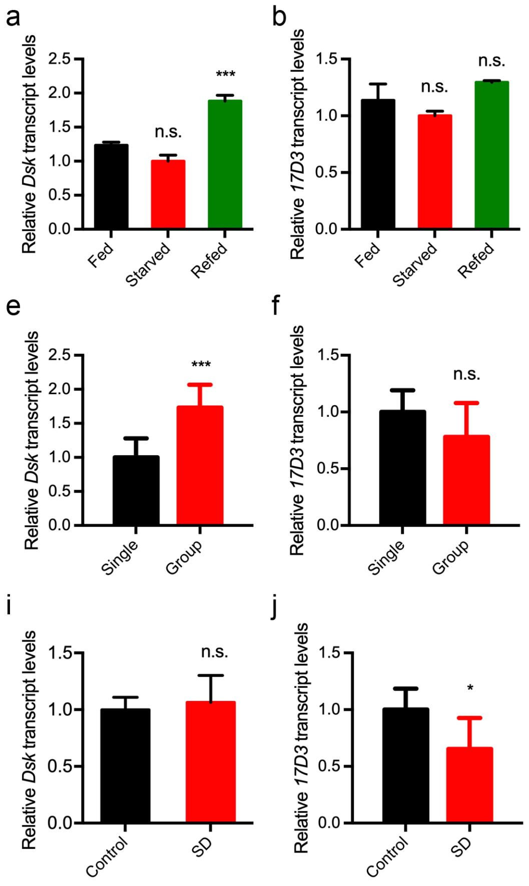

Supplementary Fig. 7 *Dsk* and *CCKLR-17D3* expression responds to different physiological conditions. **a**, **b** Refed after starvation increases *Dsk* mRNA transcript but has no impact on *CCKLR-17D3* expression. **c**, **d** Aging increases *Dsk* mRNA transcript but decreases *CCKLR-17D3* expression. **e**, **f** Group housing increases *Dsk* mRNA transcript but has no impact on *CCKLR-17D3* expression. **g**, **h** Activation of P1 neurons increases *Dsk* mRNA transcript but has no impact on *CCKLR-17D3* expression. **i**, **j** Sleep-deprivation (SD) has no impact on *Dsk* mRNA transcript but decreases *CCKLR-17D3* expression. n = 10-12 based on three replicates for each. For **a**-**d**, ***$p < 0.001$, **$p < 0.01$, n.s., not significant by One-way ANOVA with post hoc Dunn's multiple comparisons test. For **e**-**j**, ***$p < 0.001$, *$p < 0.05$, n.s., not significant by unpaired t test. Error bars indicate SEM. Source data are provided as a

Source Data file.

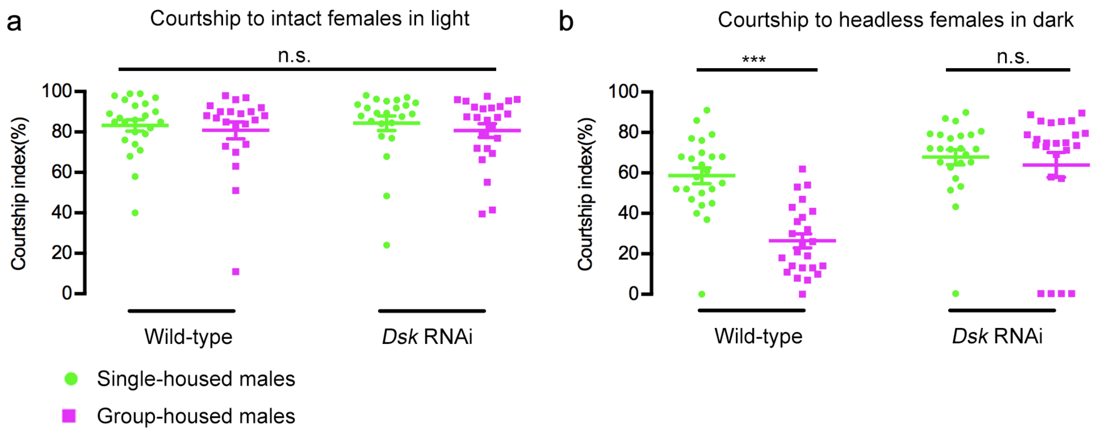

Supplementary Fig. 8 Group-housing reduces male courtship in a DSK-dependent manner. **a** Courtship towards intact females in light are not affected by group-housing in either wild-type males or *R57C10-GAL4/UAS-DskRNAi* males. *n* = 20, 22, 23, 24 respectively. **b** Group-housing reduces male courtship towards headless females in dark in wild-type males, while knock-down of *Dsk* expression eliminates such inhibitory effect. *n* = 24 for each, ***$p < 0.001$, n.s., not significant, One-way ANOVA with post hoc Dunn's multiple comparisons test. Error bars indicate SEM. Source data are provided as a Source Data file.

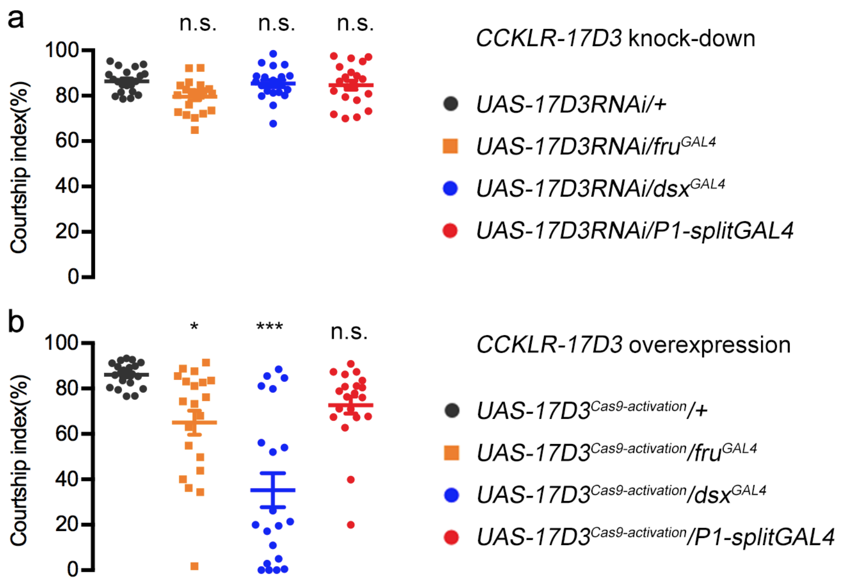

Supplementary Fig. 9 *CCKLR-17D3* inhibits male courtship in many fru^M and/or *dsx* neurons. **a** Knocking down *CCKLR-17D3* expression in all fru^M neurons, all *dsx* neurons or a subset of P1 neurons does not affect male courtship. **b** Overexpression of *CCKLR-17D3* in all fru^M neurons or all *dsx* neurons, but not a subset of P1 neurons alone, reduces male courtship. $n = 20$ for each, $*p < 0.05$, $***p < 0.001$, n.s., not significant, One-way ANOVA with post hoc Dunn's multiple comparisons test. Error bars indicate SEM. Source data are provided as a Source Data file.

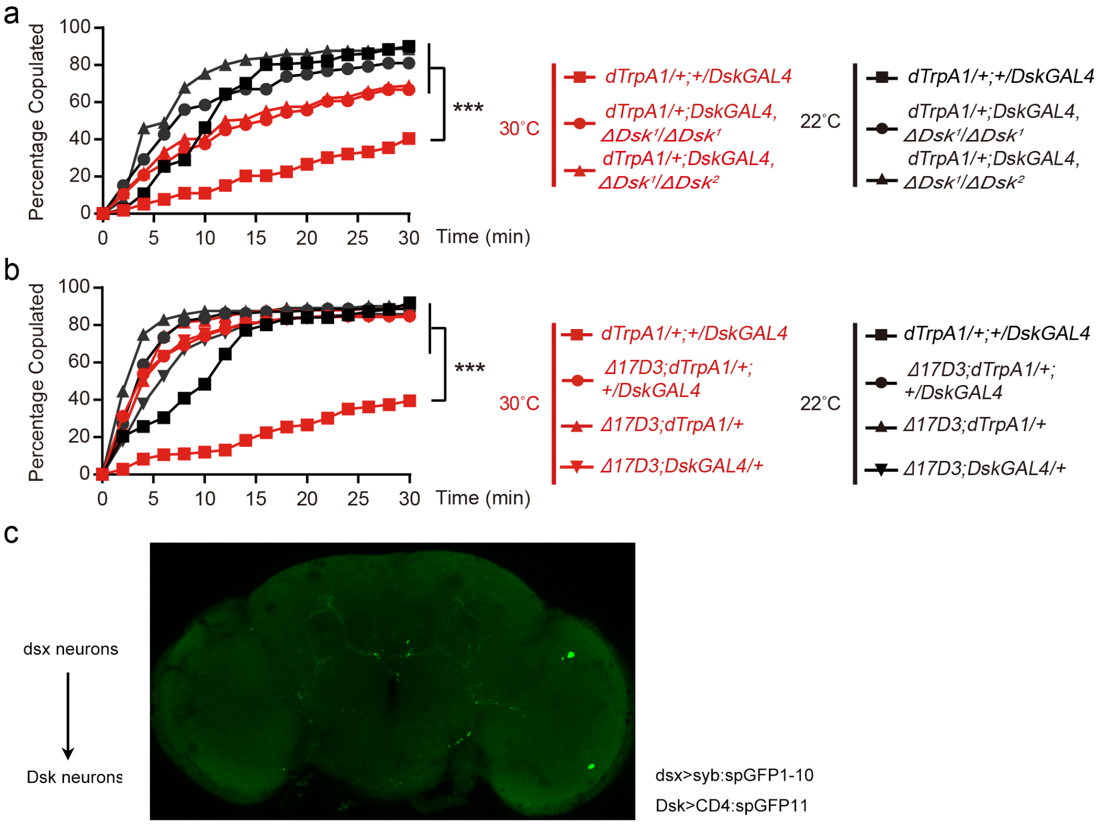

Supplementary Fig. 10 DSK/CCKLR-17D3 signaling regulates female receptivity. **a** Inhibition of female receptivity by activating *DskGAL4* neurons depends on DSK peptides. n = 120-180 for each. ***$p < 0.001$, chi-square test. **b** Inhibition of female receptivity by activating *DskGAL4* neurons depends on CCKLR-17D3. n = 108-190 for each. ***$p < 0.001$, chi-square test. **c** Synaptic transmission from *dsx* neurons to *Dsk* neurons as revealed by syb-GRASP signals. Representative of 5 female brains. Source data are provided as a Source Data file.

Supplementary Table 1. Activation of *DskGAL4* neurons does not promote ejaculation in isolated males.

Genotype	Percentage of males ejaculate (total number)	
	22˚C	30˚C
UAS-dTrpA1/+; DskGAL4/+	0% (12)	0% (24)
UAS-dTrpA1/+; fru^{GAL4}*/+*	0% (23)	100% (20)
UAS-dTrpA1/+; CrzGAL4/+	0% (21)	50% (28)
UAS-dTrpA1/CrzGAL4	0% (23)	75% (24)
