# Supplementary material for: Drosulfakinin signaling in fruitless circuitry antagonizes P1 neurons to regulate sexual arousal in Drosophila
Source: Nat Commun. 2019 Oct 18;10:4770. doi: 10.1038/s41467-019-12758-6 (PMC6800437; doi:10.1038/s41467-019-12758-6)
Supplement: Supplementary file 2 — Description of Additional Supplementary Files [file 41467_2019_12758_MOESM2_ESM.docx]

**Description of Additional Supplementary Files**

**Supplementary Movie 1**. An example episode of a *UAS-dTrpA1/+; DskGAL4/+* male with a wild-type female at 22˚C, followed by an episode of the same genotype at 30˚C.

**Supplementary Movie 2.** About thirty males of each genotype, *UAS-dTrpA1/+; DskGAL4/+* (the 2 vials on the left), *DskGAL4/+* (2nd from right), and *UAS-dTrpA1/+* (far right), were housed together at 22˚C for 5 days and then moved to 30˚C. Their behaviors were video recorded 30 min after temperature shift. *UAS-dTrpA1/+; DskGAL4/+* males showed normal locomotion response to mechanic stimulation compared with the two controls.

**Supplementary Movie 3.** Registration of a single MP1a neuron and a subset of P1 neurons (labeled by *R15A01-AD; R71G01-DBD*) in a standard brain.

**Supplementary Movie 4.** Registration of a single MP1b neuron and a subset of P1 neurons (labeled by *R15A01-AD; R71G01-DBD*) in a standard brain.

**Supplementary Movie 5.** Registration of two MP3 neurons and a subset of P1 neurons (labeled by *R15A01-AD; R71G01-DBD*) in a standard brain.
