# Supplementary material for: Drosulfakinin signaling in fruitless circuitry antagonizes P1 neurons to regulate sexual arousal in Drosophila
Source: Nat Commun. 2019 Oct 18;10:4770. doi: 10.1038/s41467-019-12758-6 (PMC6800437; doi:10.1038/s41467-019-12758-6)
Supplement: Supplementary file 9 — Reporting Summary [file 41467_2019_12758_MOESM9_ESM.pdf]

nature research

Corresponding author(s): Yufeng Pan

Last updated by author(s): Jul 29, 2019

# Reporting Summary

Nature Research wishes to improve the reproducibility of the work that we publish. This form provides structure for consistency and transparency in reporting. For further information on Nature Research policies, see Authors & Referees and the Editorial Policy Checklist.

## Statistics

For all statistical analyses, confirm that the following items are present in the figure legend, table legend, main text, or Methods section.

| n/a | Confirmed | |
|---|---|---|
| ☐ | ☒ | The exact sample size (*n*) for each experimental group/condition, given as a discrete number and unit of measurement |
| ☐ | ☒ | A statement on whether measurements were taken from distinct samples or whether the same sample was measured repeatedly |
| ☐ | ☒ | The statistical test(s) used AND whether they are one- or two-sided<br>*Only common tests should be described solely by name; describe more complex techniques in the Methods section.* |
| ☒ | ☐ | A description of all covariates tested |
| ☐ | ☒ | A description of any assumptions or corrections, such as tests of normality and adjustment for multiple comparisons |
| ☐ | ☒ | A full description of the statistical parameters including central tendency (e.g. means) or other basic estimates (e.g. regression coefficient) AND variation (e.g. standard deviation) or associated estimates of uncertainty (e.g. confidence intervals) |
| ☐ | ☒ | For null hypothesis testing, the test statistic (e.g. *F*, *t*, *r*) with confidence intervals, effect sizes, degrees of freedom and *P* value noted<br>*Give P values as exact values whenever suitable.* |
| ☒ | ☐ | For Bayesian analysis, information on the choice of priors and Markov chain Monte Carlo settings |
| ☒ | ☐ | For hierarchical and complex designs, identification of the appropriate level for tests and full reporting of outcomes |
| ☒ | ☐ | Estimates of effect sizes (e.g. Cohen's *d*, Pearson's *r*), indicating how they were calculated |

*Our web collection on statistics for biologists contains articles on many of the points above.*

## Software and code

Policy information about availability of computer code

| | |
|---|---|
| Data collection | Video recording: Sony FDR-AX40; sleep: DAM System 308; confocal: ZEN 2012; qPCR: Light Cycler 96 |
| Data analysis | Courtship: LifeSong X; sleep: DAMFile Scan107 and Matlab-R2018a; locomotion: Viewpoint Application Manager-Zebralab, Microsoft Excel; confocal: ImageJ; statistics and figure drawing: Prism 7. |

For manuscripts utilizing custom algorithms or software that are central to the research but not yet described in published literature, software must be made available to editors/reviewers. We strongly encourage code deposition in a community repository (e.g. GitHub). See the Nature Research guidelines for submitting code & software for further information.

## Data

Policy information about availability of data

All manuscripts must include a data availability statement. This statement should provide the following information, where applicable:
- Accession codes, unique identifiers, or web links for publicly available datasets
- A list of figures that have associated raw data
- A description of any restrictions on data availability

All data generated or analyzed during this study are included in the manuscript and its supplementary information files. All other relevant data supporting the findings of this study (e.g. transgenic flies) are available from the corresponding author upon reasonable request.

# Field-specific reporting

Please select the one below that is the best fit for your research. If you are not sure, read the appropriate sections before making your selection.

☒ Life sciences ☐ Behavioural & social sciences ☐ Ecological, evolutionary & environmental sciences

For a reference copy of the document with all sections, see [nature.com/documents/nr-reporting-summary-flat.pdf](nature.com/documents/nr-reporting-summary-flat.pdf)

# Life sciences study design

All studies must disclose on these points even when the disclosure is negative.

| | |
|---|---|
| Sample size | Sample size for male courtship, sleep or locomotion test is > 20, for female receptivity is > 50, for tissue staining and quantification > 7. All samples sizes are indicated in each figure legend. |
| Data exclusions | No data were excluded from the analyses. |
| Replication | All data presented are representative of at least two independent experiments as indicated in the method part of statistics. |
| Randomization | Randomization was irrelevant for the design of this study. All the experimental groups were allocated based on genotypes, and appropriate control groups were assayed in parallel. |
| Blinding | Investigators were not blinded during data collection or analysis. |

# Reporting for specific materials, systems and methods

We require information from authors about some types of materials, experimental systems and methods used in many studies. Here, indicate whether each material, system or method listed is relevant to your study. If you are not sure if a list item applies to your research, read the appropriate section before selecting a response.

## Materials & experimental systems

| n/a | Involved in the study | |
|---|---|---|
| ☐ | ☒ | Antibodies |
| ☒ | ☐ | Eukaryotic cell lines |
| ☒ | ☐ | Palaeontology |
| ☐ | ☒ | Animals and other organisms |
| ☒ | ☐ | Human research participants |
| ☒ | ☐ | Clinical data |

## Methods

| n/a | Involved in the study | |
|---|---|---|
| ☒ | ☐ | ChIP-seq |
| ☒ | ☐ | Flow cytometry |
| ☒ | ☐ | MRI-based neuroimaging |

## Antibodies

| | |
|---|---|
| Antibodies used | Primary antibodies: rabbit anti-GFP (Invitrogen A11122, 1:1000), mouse anti-Bruchpilot (Developmental 722 Studies Hybridoma Bank nc82, 1:30), rabbit anti-DSK (generated in this study, see methods, 1:100).<br>Secondary antibodies used: goat anti-mouse IgG conjugated to Alexa 488 (1:500) or Alexa 555 (1:500) and goat anti-rabbit IgG conjugated to Alexa 488 (1:500) or Alexa 555 (1:500) (Thermo Fisher Scientific). |
| Validation | All antibodies except for anti-DSK antibody generated in this study were widely used and marketed commercially as validated products. Anti-DSK antibody was validated by its staining of previously described DSK cells in the fly brain. |

## Animals and other organisms

Policy information about [studies involving animals](studies involving animals); [ARRIVE guidelines](ARRIVE guidelines) recommended for reporting animal research

| | |
|---|---|
| Laboratory animals | Drosophila melanogaster. |
| Wild animals | The study did not involve wild animals. |
| Field-collected samples | The study did not involve samples collected from the field. |
| Ethics oversight | No ethical oversight was required as no vertebrate animals were involved in the research, and no ethical oversight of the experiments was required by our institution. |

Note that full information on the approval of the study protocol must also be provided in the manuscript.
